# The Association between Dietary Variety and Physical Frailty in Community-Dwelling Older Adults

**DOI:** 10.3390/healthcare9010032

**Published:** 2021-01-01

**Authors:** Yuto Kiuchi, Hyuma Makizako, Yuki Nakai, Kazutoshi Tomioka, Yoshiaki Taniguchi, Mika Kimura, Hiroaki Kanouchi, Toshihiro Takenaka, Takuro Kubozono, Mitsuru Ohishi

**Affiliations:** 1 Department of Health Science, Graduate School of Health Sciences Kagoshima University, Kagoshima 890-8544, Japan; yuto.kch55@gmail.com (Y.K.); reha_tommy@yahoo.co.jp (K.T.); p.taniguchi0601@gmail.com (Y.T.); 2 Department of Physical Therapy, Faculty of Medicine, School of Health Sciences, Kagoshima University, Kagoshima 890-8544, Japan; nakai@health.nop.kagoshima-u.ac.jp; 3 Tarumizu Municipal Medical Center Tarumizu Chuo Hospital, Kagoshima 891-2124, Japan; takenaka@tarumizumh.jp; 4 Department of Physical Therapy, Kagoshima Medical Professional College, Kagoshima 891-0133, Japan; 5 Center for Health Promotion, International Life Sciences Institute, Tokyo 102-0083, Japan; mika-kimura@ilsijapan.org; 6 Department of Clinical Nutrition, School of Rehabilitation, College of Health and Human Science, Osaka Prefecture University, Osaka 583-8555, Japan; kano@rehab.osakafu-u.ac.jp; 7 Department of Cardiovascular Medicine and Hypertension, Graduate School of Medical and Dental Sciences, Kagoshima University, Kagoshima 890-8520, Japan; kubozono@m.kufm.kagoshima-u.ac.jp (T.K.); ohishi@m2.kufm.kagoshima-u.ac.jp (M.O.)

**Keywords:** physical frailty, diet variety, aging

## Abstract

The aim of this cross-sectional study was to examine the association between diet variety and physical frailty in community-dwelling older adults. Data of 577 older adults (mean age: 74.0 ± 6.3 years, women: 62.5%) were analyzed. Diet variety was assessed using the Food Frequency Score (FFS) (maximum, 30 points). The FFS assessed the one-week consumption frequency of ten foods (meat, fish/shellfish, eggs, milk & dairy products, soybean products, green & yellow vegetables, potatoes, fruits, seafood, and fats & oil). Physical frailty was assessed using Fried’s component (slowness, weakness, exhaustion, low physical activity, and weight loss). The participants were classified into frail, pre-frail, and non-frail groups. The prevalence of physical frailty was 6.6%. This study found significant associations between physical frailty and low FFS after adjusting for covariates (odds ratio (OR) 0.90, 95% confidence interval (CI) 0.84–0.97, *p* < 0.01). The optimal cutoff point of the FFS for physical frailty was ≤16 points. FFS lower than the cutoff point were significantly associated with physical frailty after adjusting for covariates (OR 3.46, 95% CI 1.60–7.50, *p* < 0.01). Diet variety assessed using the FFS cutoff value of ≤16 points was related to the physical frailty status in community-dwelling older adults.

## 1. Introduction

Physical frailty is concerned with a decline in physical function and a state of vulnerability to poor resolution of homeostasis after a stressful event and is a consequence of a cumulative decline of physiological systems during a lifetime [1,2]. Physical frailty leads to adverse health outcomes such as mortality, hospitalization, fall, and disability [1,2,3]. In order to extend healthy life expectancy, effective prevention and improvement strategies are required [1], and modifiable risk factors of physical frailty need to be identified.

Nutritional factors are modifiable risk factors for physical frailty [4]. Nutritional factors affect muscle homoeostasis, which is responsible for maintaining a balance between new muscle cell formation, hypertrophy, and protein loss. Thus, malnutrition causes progressive losses of skeletal muscle mass and strength [2]. Additionally, malnutrition has been confirmed to be associated with poor physical function and quality of life in older adults [5,6]. Therefore, preventing malnutrition is a key component in preventing frailty. 

The consumption of particular food and nutrients as well as diet quality are important for preventing adverse health effects [7,8]. Additionally, poor diversity in food leads to increased mortality [9,10] and may affect physical and cognitive functions in older adults [11,12]. These factors suggest that improvements in food diversity may lead to the prevention of and improvement in the geriatric syndrome. In particular, higher adherence to dietary scores (e.g., Mediterranean diet score (MDS), Diet Quality Index (DQI-I) can benefit from preventive on-set sarcopenia and physical frailty in older adults [13,14]. MDS and DQI-I were found in olive oil, fruits, vegetables, fish, and nuts, which were associated with lower levels of physical frailty [13]. Three patterns of DQI-I, particularly vegetable-fruits and snacks-drinks-milk products, were associated with lower rates of sarcopenia in Chinese men [14]. Furthermore, several studies have reported that the Japanese dietary pattern has been correlated with an overall reduction in adverse outcomes in Japan [15,16,17]. The eight-component Japanese diet index consists of the following components: high intake of rice, miso soup, seaweeds, pickles, green tea, yellow vegetables and fish, and low intake of beef and pork. A high JDI-8 score was associated with decreased risk of mortality in Japan [15]. Furthermore, a previous study reported that high adherence to Japanese traditional food was associated with lower rates of sarcopenia [18]. However, few studies are available on the association between Japanese dietary patterns and physical frailty. The Food Frequency Score (FFS) is a tool used to measure food diversity based on the Japanese dietary pattern [19,20]. FFS can easily and accurately evaluate the intake frequency of 10 foods per week [20]. If the association between FFS and physical frailty can be clearly established, the contribution of the diversity in food consumed in FFS to prevent or improve frailty can be identified. We hypothesized that FFS had an impact on frailty, and older adults with low FFS suffered from physical frailty compared with older adults with high FFS. Thus, this cross-sectional study aimed to examine the association of physical frailty with diet variety using the FFS, and to calculate optimal cutoff values of FFS for physical frailty among community-dwelling older adults.

## 2. Materials and Methods

### 2.1. Participants

**Figure 1:**
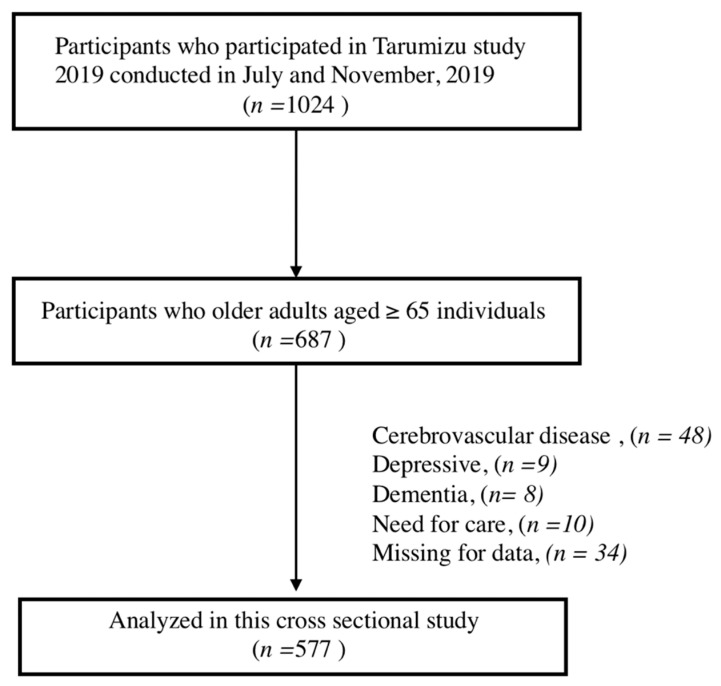
Participant inclusion criteria flow diagram.

This cross-sectional study analyzed data from the Tarumizu Study, which included a community-based health survey conducted in collaboration with Kagoshima University (Faculty of Medicine), Tarumizu City Office, and Tarumizu Chuo Hospital since 2017 [21,22,23]. The individuals were recruited from Tarumizu city, Japan, which is a residential suburb of Kagoshima city. We mailed reply-paid postcards to recruit residents in Tarumizu city who were aged 40 years or older at the time of examination (2019), and we also recruited residents through local newspaper advertisements and community campaigns. The current study used data from a health check survey conducted from June to December 2019. From a total of 1024 individuals aged ≥ 40 years in the Tarumizu study in 2019, we extracted the data of 687 of older adults aged ≥ 65 years to evaluate the association between physical frailty and diet diversity. The exclusion criteria were histories of diagnoses of severe neurological diseases (e.g., dementia and cerebrovascular disease), depression, having received certification for long-term care, and having missing data. Finally, data from 577 community-dwelling older adults (mean age, 74.0 ± 6.3 years; women, 62.5%) were analyzed (Figure 1). This study was approved by the Kagoshima University (Faculty of Medicine) Ethics Committee (Ref No.170351), and informed consents were obtained from all the participants prior to their inclusion in the study.

### 2.2. Measures

#### 2.2.1. Physical Frailty

We operationalized physical frailty using Fried’s components: slowness, weakness, exhaustion, low physical activity, and weight loss [24,25]. Accordingly, the participants were classified into frail (three or more characteristics), pre-frail (one or two characteristics), and non-frail groups. Slowness was defined using a walking speed cutoff of <1.0 m/s [26]. The participants walked and passed the location where the stand was installed, and the walking time was automatically measured using a photoelectric sensor-type gait measuring instrument (YW; YAGAMI INC., Aichi, Japan). They were asked to walk 14 m (divided into two 2.0 -m -long end zones, and a 10 -m -long middle zone) at their usual walking speed, after which their walking speed was calculated (m/s). Weakness was defined according to a sex-specific maximum grip strength cutoff (<26 kg for men and <18 kg for women) [27]. Dominant handgrip was assessed using a Smedley-type handheld dynamometer (GRIP-D; Takei, Niigata, Japan) [22]. Exhaustion was assessed using questions from the Kihon checklist; for example, the question, “In the last two weeks, have you felt tired without reason?” [28] was used to assess endurance. A response of “Yes” indicated exhaustion. Furthermore, physical activity was assessed using the following questions: (1) “Do you engage in moderate levels of physical exercise or sports aimed at health?” and (2) “Do you engage in low levels of physical exercise aimed at health?” Responses of “no” to both questions indicated low physical activity [26]. Additionally, weight loss was examined with the following question: “Have you lost 2 kg or more in the last 6 months?” A response of ‘yes’ to this question indicated weight loss [28].

#### 2.2.2. Assessment of Diet Variety

Diet variety was assessed using the FFS [19,20]. FFS was self-reported, and it assessed the one-week consumption frequency of ten food groups on the day of participation in the study. The FFS includes meat, fish/shellfish, eggs, milk and dairy products, soy bean products, green and yellow vegetables, potatoes, fruits, seafood, and fats and oil. FFS allotted to each food category ranged from 0 to 3 points based on the following responses: (1) eat almost every day (3 points), (2) eat three or four days a week (2 points), (3) eat one or days a week (1 point), and (4) hardly ever eat (0 points). The FFS was the sum of the scores; lower scores indicated low diet variety (range 0–30) [20]. 

#### 2.2.3. Demographic Variables and Covariates

Demographic data, assessed by face-to-face interviews, included age, sex, education, medical history with chronic disease (e.g., hypertension, diabetes, and hyperlipidemia), and total medications. Participants’ medical conditions, including their history and medication, were assessed using interviews by licensed doctors or nurses. The examined covariates were as follows: age, sex, education, total medications, and body mass index (BMI). 

#### 2.2.4. Statistical Analysis

We conducted all analyses using SPSS ver. 26.0 (IBM Japan, Tokyo, Japan). The Mantel–Haenszel test was used to compare the trends for categorical values among the frailty statuses (non-frailty, pre-frailty, and frailty). A one-way analysis of variance was used for continuous measures to examine differences in frailty statuses. 

A multivariate logistic regression analysis was used to examine the association between physical frailty and FFS, with physical frailty as the dependent variable. The first model (Model 1) was adjusted for age and sex. We then used a multi-adjusted model including adjustments for age, sex, BMI, education, and polypharmacy (Model 2). Polypharmacy was defined as six or more medications using cutoffs drawn from a previous study [29]. Additionally, the associations between physical frailty subitems and FFS were assessed using multivariate logistic regression. The adjusted model in the multivariate logistic regression analysis included age and sex as covariates. Odds ratios (ORs) and 95% confidence intervals (95% CIs) for incidents related to physical frailty were calculated. Additionally, receiver-operating characteristic (ROC) curves were used to determine FFS cutoff points for physical frailty. Cutoff points were determined using the Youden Index, and were calculated by maximizing the sensitivity and specificity of physical frailty. The Youden index defined the maximum vertical distance between the ROC curve and the diagonal or chance line [30]. A value of less than 0.05 was considered statistically significant.

## 3. Results

### 3.1. Characteristics of the Participants According to Frail Status

Table 1 describes the participants’ demographic characteristics and FFS. The prevalence of pre-frailty and physical frailty was 46.9% and 6.6%, respectively. Compared with the participants in the robust and pre-frail groups, those with physical frailty were significantly older, less educated, and had more prescribed medications and lower FFS.

### 3.2. Association between Physical Frailty and Diet Score

The results of the multivariate logistic regression analyses are described in Table 2. In the first model (model 1), adjusted for age and sex, low FFS (OR 0.89, 95% CI 0.83–0.96, *p* < 0.01) were significantly associated with physical frailty. In Model 2, adjusted for age, sex, BMI, education, and polypharmacy, low FFS (OR 0.90, 95% CI 0.84–0.97, *p* < 0.01) were significantly associated with physical frailty. 

The results of the multivariate logistic regression analyses are described in Table 3. In this model, adjusted for age and sex, low FFS were significantly associated with several physical frailty subitems including weight loss (OR 0.95, 95% CI 0.91–1.00, *p* = 0.04), physical inactivity (OR 0.91, 95% CI 0.87–0.96, *p* < 0.01), weakness (OR 0.93, 95% CI 0.88–0.97, *p* < 0.01), and slowness (OR 0.88, 95% CI 0.83–0.94, *p* < 0.01). However, low FFS were not significantly associated with exhaustion. 

An ROC curve analysis was conducted to calculate the optimal cutoff point of FFS for physical frailty. In this study, the optimal cutoff point of FFS for physical frailty was found to be ≤16 from the Youden Index, with sensitivity and specificity of 0.826 and 0.395, respectively.

FFS lower than the cutoff point were significantly associated with physical frailty even after adjusting for age and sex (Model 1: OR 3.87, 95% CI 1.85–8.07, *p* < 0.01). In Model 2 adjusted for age, sex, BMI, education, and polypharmacy, values lower than the cutoff point of FFS (OR 3.46, 95% CI 1.60–7.50, *p* < 0.01) remained significantly associated with physical frailty (Table 4).

## 4. Discussion

The present cross-sectional study revealed that older adults with low FFS had higher prevalence of frailty than those with high FFS. The optimal cutoff point for physical frailty was ≥16. In reality, people do not consume isolated nutrients, but they eat meals comprising complex combinations of various nutrients. Therefore, a single-nutrient approach is insufficient to understand the complicated interactions among nutrients [8]. Several studies have reported that poor diet diversity had negative impacts on health such as mortality risk, chronic disease, and loss of physical function in older adults [14,31,32,33]. Thus, it is inferred that consuming a variety of foods benefits health outcomes in older adults more than individual foods. The FFS included the frequencies of consumptions of meat, fish/shellfish, eggs, milk, and soybean products, which are all rich in protein [34]. Ingesting high-protein foods may prevent physical frailty and enhance muscle function in older adults [35,36,37]. Meat and fish/shellfish, i.e., animal based protein, contain high amounts of protein digestibility corrected amino acids and branched chain amino acids; therefore, their consumption encourages muscular anabolic processes [38]. Furthermore, a previous study reported that an inadequate vitamin D level was associated with poor physical function and muscle strength [39]. Fish are sources of protein and vitamin D [40]. Soybean products contain plant-based proteins, and the consumption of plant-based proteins was associated with reduced losses of muscle mass [41,42,43]. Additionally, high intake of protein was associated with physical frailty, but the source and quality of protein were not associated with physical frailty [44]. The FFS comprises 10 foods, of which 5 foods contain protein. It was inferred that consuming combinations of foods that are protein-rich had an impact on physical frailty in older adults. Further, green and yellow vegetables and fruits contain nutrients with antioxidant properties, such as vitamin C and vitamin E. These nutrients seem to suppress nerve cell damage and cell death caused by oxidative reactions [45,46]. Bartali et al. reported that low serum vitamin E levels were associated with poor physical functions in community-dwelling older adults [47]. Moreover, a cross-sectional study in Japan has shown that high-protein diets and high-dietary total antioxidant capacity diets were inversely associated with physical frailty [48]. Furthermore, the FFS included foods that contain nutrients with antioxidants and proteins. Thus, we believe that ingesting a combination of foods included in the FFS may help to prevent or improve physical frailty and decline in physical function. 

Previous studies have reported that FFS of 15 or lower were associated with malnutrition in home-care recipients [19]. This study calculated the optimal cutoff point of FFS for physical frailty using ROC curves, and we applied values ≥ 16. Thus, the calculated FFS optimal cutoff point was similar to the results of a previous study. This study indicated that FFS ≥16 were associated with physical frailty. The diet variety score (DVS) is an assessment tool for food diversity in Japan along with FFS [49]. The FFS contains the same 10 foods as the DVS. The DVS assessed the frequency of intake of 10 foods per week, with one point for the consumption of each food every day for a week and zero points for everything total, a total of 0 to 10 points. The DVS confirmed the associations between physical function, body composition, and higher brain function [34,49,50]. The FFS is a variant of the DVS. It can help to obtain detailed information on the frequency of 10 foods per week and can easily be used to assess food diversity. To the best of our knowledge, this is the first study to reveal the association between physical frailty and diet variety using FFS. 

The present study had several limitations. First, FFS used the Qualitive Food Frequency Questionnaire, which does not consider the quantity consumed [51]; therefore, it was difficult to estimate the quantity of food. Thus, it was unclear as to whether diet variety affects physical frailty, or the amount of food consumed affects physical frailty. Future research should take into account the amount of food consumed. Second, it was difficult to analyze FFS for each nutrient and the interactions of each nutrient. However, a previous study evaluated the association between DVS and each nutrient [52]. The association between FFS and each nutrient should be confirmed. Furthermore, in the future, studies should elucidate how FFS food interaction affects physical function of older adults. Third, in the multivariate analyses of the present study, cognitive and psychological domains were not included. In addition, other potential covariates, such as lifestyle and socioeconomic status, that are likely related to diet variety and physical frailty should be considered [53]. Fourth, the participants were not recruited randomly in the community. This study enrolled those older adults who, on their own, participated in a venue-based health check survey and were, therefore, likely to be healthy older adults, which may have led to an underestimation of physical frailty [25]. Fifth, we did not have access to the participants’ medical records which were confirmed by their physicians. Finally, the present study was a cross-sectional study; hence, it does not identify the causal effects of FFS on physical frailty. Future research should elucidate whether diet variety with FFS predicts the development of physical frailty.

## 5. Conclusions

The FFS assessed diet variety using 10 foods. This study confirmed that poor food variety as assessed by FFS was associated with physical frailty. In particular, FFS scores of 16 and lower were associated with physical frailty. This study revealed that it may be possible to prevent physical frailty by improving diet diversity using the FFS.

## Figures and Tables

**Table 1:** Characteristics of the participants (mean ± or %).

Variable	Overall	Non-Frailty	Pre-Frailty	Frailty	*p*
	(*n* = 577)	(*n* = 268)	(*n* = 271)	(*n* = 38)	
Age (years)	74.0 ± 6.3	72.4 ± 5.2	74.9 ± 6.7	78.3 ± 7.4	<0.01
Women, *n* (%)	361, (62.5%)	181, (67.5%)	152, (56.1%)	28, (73.7%)	0.02
Education (years)	11.5 ± 2.2	11.8 ± 2.0	11.4 ± 2.4	10.1 ± 1.8	<0.01
BMI, kg/m^2^	23.1 ± 3.2	23.0 ± 3.0	23.3 ± 3.3	22.4 ± 3.9	0.25
Medication (*n*/day)	3.1 ± 3.0	2.3 ± 2.6	3.6 ± 3.1	5.0 ± 3.7	<0.01
FFS	20.3 ± 4.6	21.2 ± 4.4	19.7 ± 4.7	18.4 ± 4.5	<0.01
Chronic disease, *n* (%)					
Hypertension	317, (55.0%)	135, (50.4%)	156, (57.6%)	26, (68.4%)	0.02
Diabetes mellitus	72, (12.5%)	21, (7.8%)	46, (17.0%)	5, (13.2%)	<0.01
Hyperlipidemia	162, (28.0%)	73, (27.2%)	76, (28.0%)	13, (34.2%)	0.47

Data shown as the mean ± SD or percentage. One-way analysis of variance for continuous measures and Mantel–Haenszel test for proportions. BMI, body mass index; FFS, food frequency score.

**Table 2:** Association between food frequency score and physical frailty.

Dependent Value: Presence of Physical Frailty				
Independent Variable	Model 1		Model 2	
	OR	(95% CI)	OR	(95% CI)
FFS	0.89 **	(0.83–0.96)	0.90 *	(0.84–0.97)
Age	1.11 **	(1.01–1.17)	1.09 **	(1.03–1.15)
Sex (women)	1.95	(0.89–4.26)	1.59	(0.71–3.56)
Educations			0.79 *	(0.65–0.96)
BMI			0.89 *	(0.80–0.99)
polypharmacy (vs. 6 or less medications)			1.50	(0.69–3.28)

Note: OR, odds ratio; CI; confidence interval; BMI, Body Mass Index; FFS, food frequency score; the bold type face indicates statistical significance; ** *p* < 0.01, * *p* < 0.05; Model 1: adjusted for age and sex; Model 2: adjusted for age, sex, education, body mass index, and polypharmacy.

**Table 3:** Association between food frequency score and sub-items of physical frailty.

Dependent Value: Each of Subitems of Physical Frailty										
Independent Variable	Weight Loss		Exhaustion		Physical Inactivity		Weakness		Slowness	
	OR	(95% CI)	OR	(95% CI)	OR	(95% CI)	OR	(95% CI)	OR	(95% CI)
FFS	0.95 *	(0.91–1.00)	0.98	(0.93–1.03)	0.90 **	(0.86–0.95)	0.91 **	(0.87–0.96)	0.87 **	(0.82–0.93)
Age	1.01	(0.97–1.04)	1.01	(0.98–1.05)	1.02	(0.99–1.06)	1.16 **	(1.16–1.20)	1.21 **	(1.15–1.27)
Sex (women)	0.68	(0.43–1.06)	0.88	(0.56–1.38)	0.66	(0.42–1.06)	1.72 *	(1.06–2.78)	2.11 *	(1.05–4.23)

Note: OR, odds ratio; CI; confidence interval; FFS, food frequency score; adjusted for age and sex; the bold type face indicates statistical significance; ** *p* < 0.01, * *p* < 0.05; adjusted for age and sex.

**Table 4:** Association between loss of Food frequency score and physical frailty.

Dependent Value: Presence of Physical Frailty				
Independent Variable	Model 1		Model 2	
	OR	(95% CI)	OR	(95% CI)
Food frequency score				
FFS > 17	Reference		Reference	
FFS < 16	3.87 **	(1.85–8.07)	3.46 *	(1.60–7.50)
Age	1.11 **	(1.05–1.16)	1.08 *	(1.02–1.14)
Sex (women)	2.04	(0.93–4.45)	1.69	(0.75–3.81)
Educations			0.79 *	(0.65–0.97)
BMI			0.89 *	(0.80–0.99)
polypharmacy (vs. 6 or less medications)			1.49	(0.68–3.26)

Note: OR, odds ratio; CI; confidence interval; BMI, body mass index; FFS, Food frequency score; the bold type face indicates statistical significance; ** *p* < 0.01, * *p* < 0.05; Model 1: adjusted for age and sex; Model 2: adjusted for age, sex, educations, body mass index and polypharmacy.

## Data Availability

Data sharing not applicable.
